# Oenothein B ameliorates hepatic injury in alcoholic liver disease mice by improving oxidative stress and inflammation and modulating the gut microbiota

**DOI:** 10.3389/fnut.2022.1053718

**Published:** 2022-12-12

**Authors:** Lu Xu, Wei Li, Shu-yi Chen, Xi-wen Deng, Wei-feng Deng, Guo Liu, Yun-jiao Chen, Yong Cao

**Affiliations:** ^1^Guangdong Provincial Key Laboratory of Nutraceuticals and Functional Foods, Guangdong Research Center for Engineering Technology in Bioactive Natural Products, College of Food Science, South China Agricultural University, Guangzhou, China; ^2^Institute of Biopharmaceutical and Health Engineering, Tsinghua Shenzhen International Graduate School, Shenzhen, China

**Keywords:** Oenothein B, alcoholic liver disease, oxidative stress, inflammation, gut microbiota

## Abstract

**Introduction:**

Alcoholic liver disease (ALD) is a global health problem for which there is no current food and drug administration (FDA)-approved therapy. Oenothein B (OEB) is a macrocyclic dimer ellagic tannin that possesses abundant biological activities including antioxidant, anti-inflammation, antitumor, immunomodulatory, and antimicrobial properties.

**Materials and methods:**

In this study, the hepatoprotective effect of OEB against ALD was investigated *in vivo* and *in vitro*.

**Results:**

We found that OEB treatment dramatically reduced alcohol-induced hepatic injury, as evidenced by decreased levels of aminotransferases and inflammatory biomarkers and increased antioxidant capacity in OEB-treated groups.

**Discussion:**

OEB treatment alleviated oxidative stress by upregulating the Keap1/Nrf2 signaling pathway and inhibited inflammation by downregulating the TLR4/NF-κB signaling pathway. Additionally, OEB treatment positively improved alcohol-induced intestinal microbial dysbiosis by modulating the structure and composition of gut microbiota. Interestingly, we observed the increasement of short-chain fatty acid (SCFA) producers (*Muribaculaceae*) and the decreasement of Gram-negative bacteria (*Akkermansia*) in the OEB treatment groups, which may contribute to the inhibition of hepatic oxidative stress and inflammation via the gut-liver axis. In summary, our findings indicate that OEB is a promising therapeutic strategy for preventing and treating ALD.

## Introduction

Alcoholic liver disease (ALD) is caused by long-term, excessive alcohol intake and is a serious burden to global public health (1). There are approximately 3.3 million annual deaths due to alcohol abuse, which accounts for nearly 6% of deaths worldwide (2). Alcohol consumption has been proven to disrupt the normal function of multiple organs including the liver, gastrointestinal system, and brain (3). ALD typically progresses from alcoholic steatosis to steatohepatitis, cirrhosis, and even hepatocellular carcinoma (4, 5). There is no available food and drug administration (FDA)-approved therapy for ALD currently (6), thus there is an urgent need to develop new drugs to both prevent and treat this disease.

Emerging evidence indicates that oxidative stress and inflammation are major contributors to the incidence and progression of ALD (7, 8). Alcohol metabolism can accelerate the production of reactive oxygen species (ROS) and impairs the body’s antioxidant systems. This causes hepatic oxidative stress which leads to apoptosis and necrosis (9). ROS can also activate inflammatory cells to induce inflammation, and the activation of immune cells further produce ROS and reactive nitrogen species to aggravate oxidative stress (10). Furthermore, induction of cytochrome P4502E1 (CYP2E1), an enzyme that converts ethanol into acetaldehyde and triggers generation of ROS, also plays a role in the progression of ALD (11). Additionally, interactions between members of the intestinal microbiome and liver play a major role in ALD pathogenesis. Briefly, acetaldehyde, a metabolic byproduct of alcohol, disrupts gut microbial composition, intestinal integrity, and gut barrier function. This leads to gut dysbiosis and leakage of the bacterial surface molecule lipopolysaccharide (LPS) into portal circulation (12, 13). It has been previously reported that this release of LPS activates toll-like receptors (TLRs) and nuclear factor-κB (NF-κB) which provokes inflammatory responses in the liver and eventually exacerbates ALD. Therefore, targeting oxidative stress, inflammation, and the gut microbiome are promising therapeutic strategies for the prevention and treatment of ALD.

Oenothein B (OEB) is a macrocyclic ellagitannin dimer produced by members of the plant kingdom. In 1990, OEB was first identified in the leaves of *Oenothera erythrosepala* (14), and it was subsequently found in plants from the families Onagraceae, Lythraceae, and Myrtaceae (15–17). Several previous studies have revealed that OEB possesses diverse biological properties including antioxidant, anti-inflammation, antitumor, immunomodulatory, and antimicrobial activities (18–23). Thus, OEB has many potential medicinal applications including preventing and treating human diseases. However, the effect of OEB on ALD has not yet been investigated. In this study, we used *in vitro* and *in vivo* ALD models to elucidate the hepatoprotective effect of OEB and the underlying mechanism.

## Materials and methods

### Chemicals and reagents

Oenothein B was obtained from the Guangdong Provincial Key Laboratory of Nutraceuticals and Functional Foods in Guangdong, China. A HepG2 human hepatocellular carcinoma cell line was purchased from the Stem Cell Bank of the Chinese Academy of Sciences. Male C57BL/6J mice were provided by Beijing Vital River Laboratory Animal Technology company.

Dulbecco’s Modified Eagle medium (DMEM), fetal bovine serum (FBS), antibiotics, and phosphate-buffered saline (PBS) were obtained from Gibco (Waltham, MA, USA). Pure, molecular biology grade ethanol was obtained from Chinasun Specialty Products Co, Ltd. (Jiangsu, China). Cell Counting Kit-8 (CCK-8) was purchased from Dojindo Laboratories (Tokyo, Japan). BCA, alanine aminotransferase (ALT), aspartate aminotransferase (AST), alkaline phosphatase (ALP), lactate dehydrogenase (LDH), superoxide dismutase (SOD), glutathione (GSH), catalase (CAT), malondialdehyde (MDA), reactive oxygen species (ROS), and triglyceride (TG) measurement and activity kits were purchased from Nanjing Jiancheng Bioengineering Institute (Nanjing, China). Nitric oxide (NO) measurement and activity kits were purchased from Beyotime Biotechnology (Shanghai, China). Tumor necrosis factor-α (TNF-α), interleukin-1β (IL-1β), and interleukin-6 (IL-6) enzyme-linked immunosorbent assay (ELISA) kits were provided by NeoBioscience Technology Co., Ltd. (Shenzhen, China). Cytochrome P4502E1 (CYP2E1) ELISA kits were purchased from Nanjing Jiancheng Bioengineering Institute (Nanjing, China). TRIzol reagent, PrimeScript RT Master Mix reverse transcription kit, and SYBR green PCR kit were purchased from TransGen Biotech (Beijing, China). All chemicals used in the present research were analytical grade.

### Cell culture

Human hepatic HepG2 cells are frequently used for *in vitro* hepatocyte model studies. A HepG2 human hepatocellular carcinoma cell line was cultured in DMEM containing 10% fetal bovine serum and 1% double antibody under 5% CO_2_ at 37°C. The culture media was changed throughout the study according to the cell growth status and color changes in the media.

### Cell viability

Cell growth and survival were examined using a Cell Counting Kit-8. Briefly, HepG2 cells were seeded into 96-well microplates (1 × 10^4^ cells per well) and cultivated for 24 h. Cells were then pre-treated with Oenothein B (0–320 μM) for 6 h, incubated with ethanol (500 mM) for 24 h, then the CCK-8 solution (10 μL) was added to each well and plates were kept at 37°C for 1 h. The absorbance at 450 nm of each well was measured using a microplate reader (PerkinElmer, USA). Plates were sealed with Parafilm-M^®^ throughout the experiments to avoid ethanol volatilization (24).

### Biochemical assay

HepG2 cells were seeded into 6-well plates (1 × 10^6^ cells per well) and grown for 24 h. Next, cells were pre-treated with Oenothein B (10, 20, and 40 μM) for 6 h then exposed to 500 mM of ethanol for 24 h. The cells and culture supernatant were obtained and the levels of ALT, AST, LDH, and NO in cell culture supernatant, and SOD, GSH, and MDA in cell homogenate, were detected with the corresponding commercial kits. Each experimental group consisted of three double wells.

### Measurement of cellular reactive oxygen species production

ROS production in HepG2 cells was examined using 2’,7’-dichlorofluorescein diacetate (DCFDA). Cells were cultured and treated as previously described in 2.4. Cells were washed thrice with ice-cold PBS then incubated with 10 mM of DCFDA in the dark for 30 min. A microplate reader was used to measure fluorescence at the emission of 530 nm and the excitation of 502 nm, and the intensity and area (%) of cellular ROS were scanned by confocal fluorescence microscopy (Axio observer A1, Carl Zeiss, Jena, Germany) using a 40 × objective.

### Animals and experimental protocols

All experimental procedures involving animals were approved by the Laboratory Animal Center of South China Agricultural University (SYXK 2014-0136). After 1 week of acclimation in a standard SPF environment at 40–60% humidity, 22–25°C, with 12/12 h light/dark cycles, a total of 32 male C57BL/6J mice (6–8 weeks, 20–24 g) were divided into four groups at random: (1) control group (CK), (2) ethanol model group (EtOH), (3) low dose Oenothein B group (OL), (4) high dose Oenothein B group (OH). Each group contained 8 mice. Mice were fed a normal diet for 4 weeks, during which the CK and EtOH groups were orally administered normal saline and the OL and OH groups were orally given 25 and 75 mg/kg OEB, respectively. After the 4-week feeding period, an oral dose of 50% ethanol (10 mL/kg body weight [b.w.]) was given every 12 h to induce ALD. Weight and food intake of all mice were noted twice per week. Fasting mice were sacrificed after the acute ethanol intake period and serum samples were collected, centrifuged at 4,000 rpm for 15 min, and stored at 80°C. Additionally, liver tissues were promptly acquired and weighed after sacrifice and the liver index was calculated according to the following formula:


Liverindex(%)=liverweight/finalbodyweight × 100%

A portion of each liver was transferred to a 4% paraformaldehyde solution for histological evaluation, and the remaining liver tissues were frozen in liquid nitrogen and stored at 80°C for further tests.

### Biochemical analysis of serum and liver tissues

Serum biomarkers such as ALT, AST, ALP, and TG, and hepatic antioxidant activities such as MDA, SOD, CAT, and GSH, were detected using corresponding kits. Levels of hepatic TNF-α, IL-1β, IL-6, and CYP2E1 were determined by ELISA kits following manufacturer instructions.

### Histological analysis of the liver

Liver tissues in 4% paraformaldehyde were sent to Biossci Biotechnology Co., Ltd. (Wuhan, China) for embedding, sectioning, and staining with hematoxylin and eosin (H&E) to determine steatosis and necrosis status. Additionally, oil-red O was used to stain the sections in order to examine the hepatic lipid accumulation. Tissue sections were scanned using a Nikon NI-E microscope (Nikon, Japan) and were viewed using NDP.view2 software.

### Quantitative real-time polymerase chain reaction

TRIzol reagent was used to obtain total RNA from liver tissues, then reverse transcription was carried out to generate cDNA. Next, quantitative real-time polymerase chain reaction was used to measure mRNA expression of Nrf2, Keap1, HO-1, NQO1, NF-κB, TLR4, Myd88, and CD14. Relative expression was calculated via the 2^–ΔΔCt^ method and Glyceraldehyde-3-phosphate dehydrogenase (Gapdh) was used as the reference gene. Primers were synthesized by Tsingke Biotechnology Co., Ltd. (Beijing, China) and are presented in Table 1.

**TABLE 1:** Primer sequences used for RT-qPCR.

Genes		Primer sequences (5′-3′)
Gapdh	Forward	GTGAAGGTCGGTGTGAACGGATTT
	Reverse	TGGCAACAATCTCCACTTTGCCAC
Nrf2	Forward	GGTTGCCCACATTCCCAAAC
	Reverse	GCAAGCGACTCATGGTCATC
Keap1	Forward	GCGTGGAGAGATATGAGCCA
	Reverse	CATACAGCAAGCGGTTGAGC
HO-1	Forward	AAGCCGAGAATGCTGAGTTCA
	Reverse	GCCGTGTAGATATGGTACAAGGA
Nqo1	Forward	CATCACAGGTGAGCTGAAGGA
	Reverse	ACAATATCTGGGCTCAGGCG
Nfkb1	Forward	AGACAAGGAGCAGGACAT
	Reverse	CCAGCAACATCTTCACATC
Tlr4	Forward	ATGGCATGGCTTACACCACC
	Reverse	GAGGCCAATTTTGTCTCCACA
Myd88	Forward	AGAACAGACAGACTATCGGCT
	Reverse	CGGCGACACCTTTTCTCAAT
Cd14	Forward	GGAAGCCAGAGAACACCATC
	Reverse	CCAGAAGCAACAGCAACAAG

https://www.jianguoyun.com/c/sd/15f9fbc/7cc501fbcf0150c2.

### Mouse gut microbiota sequencing and analysis

Fresh fecal samples from the CK, EtOH, and OEB treated groups (*n* = 3) were collected in RNase-free freezing tubes and stored at −80°C. The total genomic DNA was obtained using the cetyltrimethylammonium bromide (CTAB) method. To analyze the gut microbiome composition, bacterial 16S rRNA hypervariable regions V3-V4 were amplified by PCR using the universal primers 338F (5-ACTCCTACGGGAGGCAGCA-3) and 806R (5-GGACTACHVGGGTWTCTAAT-3). Next, PCR products were checked by 2% agarose gel electrophoresis, purified using a PCR purification Kit (Qiagen, Barcelona, Spain), and pooled in equimolar ratios. The Illumina NovaSeq platform was used to sequence the amplicons at the Beijing Novogene Technology Co., Ltd. Raw data were merged and filtered and Amplicon Sequence Variants (ASVs) were obtained using the DADA2 method. These ASVs were then annotated to the genus level and α-diversity, β-diversity, community composition, and Spearsman correlation analyses were performed using QIIME2 software.

## Results

### Oenothein B alleviates ethanol-induced cytotoxicity of HepG2 cells

**FIGURE 1:**
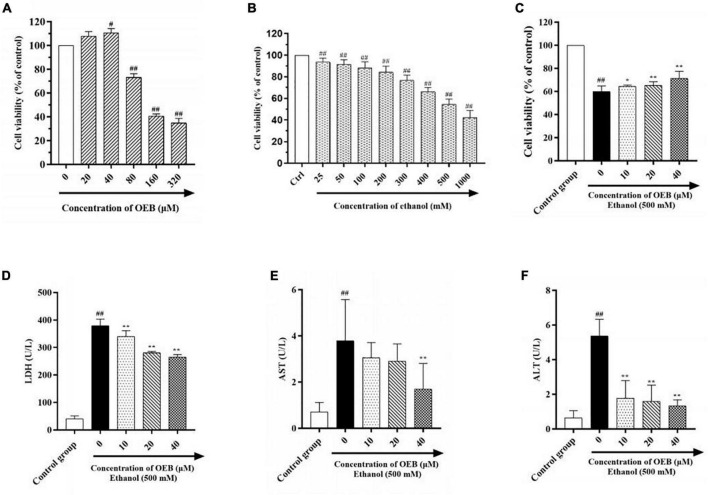
OEB alleviated ethanol induced cytotoxicity in HepG2 cells. **(A)** Cells were treated with OEB (0–320 μM) for 24 h. **(B)** Cells were treated with ethanol (0–1,000 mM) for 24 h. **(C)** Cells were pretreated with OEB (10–40 μM) for 6 h, followed by ethanol (500 mM) for 24 h. The levels of LDH **(D)**, AST **(E)**, and ALT **(F)** released by cells were detected from the culture supernatant. Data represents mean ± SD (*n* = 5). **^#^***P* < 0.05 and **^##^***P* < 0.01 with respect to the control group; *P < 0.05 and **P < 0.01 with respect to the model group.

In order to determine therapeutic concentrations of OEB, we measured the viability of HepG2 cell treated with OEB at gradient concentrations (from 0 to 320 μM) for 24 h. As shown in Figure 1A, OEB at concentrations below 40 μM had no significant effect on the cell viability, however, when the OEB concentration was over 80 μM, the cell viability decreased remarkably. These results indicate that relatively low doses of OEB may exert cytoprotective effects, therefore OEB concentrations of 10, 20, and 40 μM were used in subsequent experiments.

To establish the optimal ethanol concentration to induce the alcoholic HepG2 cell model, HepG2 cells were exposed to a range of ethanol concentrations (25–1,000 mM) for 24 h and cell viability was assessed using a CCK-8 kit. As shown in Figure 1B, cell survival decreased in a dose-dependent manner following ethanol exposure; at 500 mM of ethanol, cell viability was reduced by half (54.50 ± 4.81%). This ethanol concentration can be observed in alcoholics, therefore 500 mM of ethanol was selected to induce our model in the following studies.

To evaluate the hepatoprotective capacity of OEB, HepG2 cells were pre-treated with OEB (10, 20, or 40 μM) for 6 h, and then exposed to 500 mM of ethanol for 24 h. Cell viability significantly decreased following ethanol exposure (*P* < 0.01), however the pre-treatment of OEB in a dose-dependent manner restored the cell viability (*P* < 0.05) (Figure 1C). This indicates that OEB can protect from ethanol-induced cytotoxicity in hepatocytes.

Levels of hepatotoxicity biomarkers LDH, ALT, and AST correlate with hepatocyte membrane integrity and organelle damage (25). Thus, to further evaluate the protective effect of OEB, LDH, ALT, and AST levels in each experimental group were quantified. As shown in Figures 1D–F, ethanol exposure significantly increased LDH, ALT, and AST levels relative to the control group (*P* < 0.01). OEB pre-treatment prevented these elevations, with 40 μM of OEB demonstrating the most notable effect by maintaining LDH, ALT, and AST levels at 264.95 ± 9.38, 1.33 ± 0.35, and 1.70 ± 1.11 U/L (*P* < 0.01), respectively. Taken together, these results indicate that OEB has a hepatoprotective effect as it efficiently alleviates ethanol-induced cellular toxicity.

### Oenothein B attenuates ethanol-induced oxidative stress and inflammation of HepG2 cells

**FIGURE 2:**
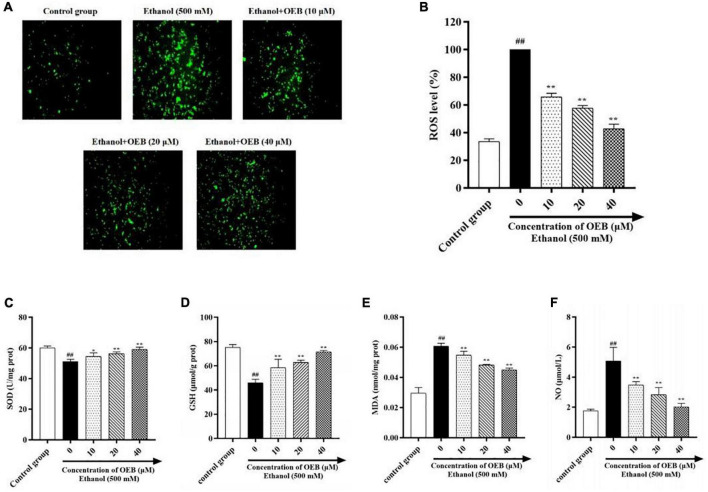
OEB suppressed ethanol-induced oxidative stress in HepG2 cells. Cells were pretreated with OEB (10, 20, and 40 μM) for 6 h, followed by ethanol (500 mM) for 24 h. **(A)** ROS levels in HepG2 cells monitored by confocal fluorescence microscopy imaging of DCF fluorescence intensity. **(B)** ROS levels were evaluated in HepG2 cells. The levels of **(C)** SOD, **(D)** GSH, and **(E)** MDA in cell homogenate and **(F)** NO in cell supernatant were evaluated. Data represents mean ± SD (*n* = 5). ^##^*P* < 0.01 with respect to the control group; *P < 0.05 and **P < 0.01 with respect to the model group.

To assess the effect of OEB on hepatic oxidative stress, ROS generation in HepG2 cells was measured using the fluorescent ROS probe DCFA and confocal microscopy. Acute ethanol exposure induced ROS production in cells, and this increased ROS production was inhibited by pre-treatment with OEB (*P* < 0.01) (Figures 2A,B). We next measured cellular levels of the antioxidant enzymes superoxide dismutase (SOD) and glutathione (GSH), both of which protect cells by scavenging excessive ROS. Ethanol treatment significantly decreased SOD and GSH levels and increased levels of malondialdehyde (MDA), a product of lipid peroxidation and a biomarker of oxidative stress. Pre-treatment with OEB increased antioxidant levels and reduced MDA (Figures 2C–E), indicating OEB may protect against lipid peroxidation. To determine the effect of OEB pre-treatment on ethanol-induced inflammation, we assessed the level of nitric oxide (NO), which was common pro-inflammatory molecule related to liver injury, in cell supernatants. OEB pre-treatment significantly reversed the elevation of NO levels induced by ethanol exposure (Figure 2F). Collectively, these results indicate that OEB reduces both hepatic oxidative stress and inflammation.

### Oenothein B improves ethanol-induced hepatic function changes and pathological alterations in alcoholic liver disease mice

**FIGURE 3:**
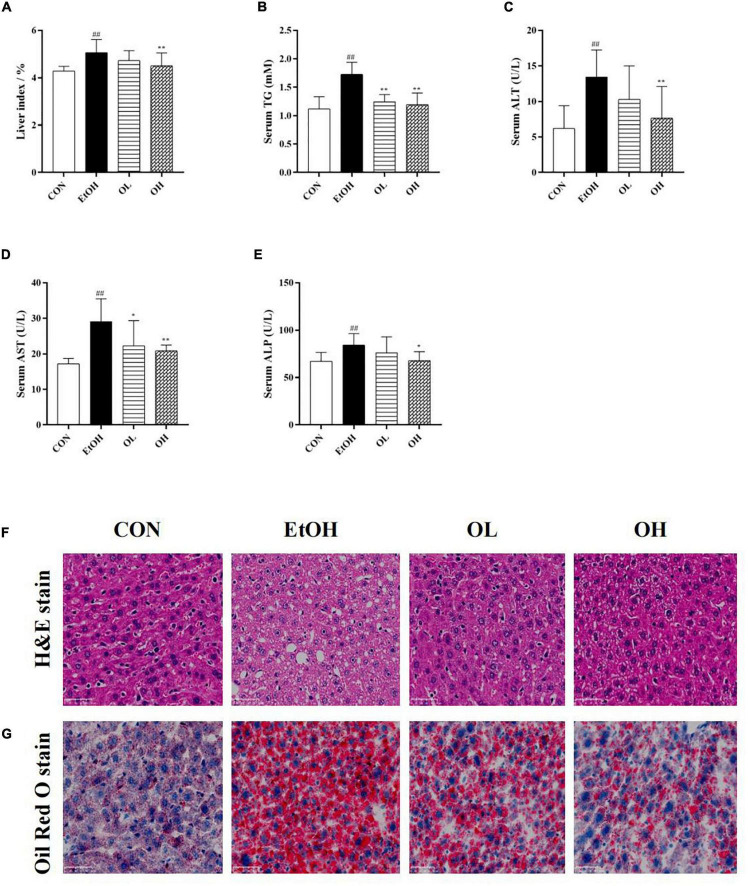
Effect of OEB treatment on hepatic basic indices and histopathological alterations of ALD mice basic indices of the mice. **(A)** Effect of OEB on liver index of mice. **(B)** Effect of OEB on hepatic triglyceride levels. **(C,D)** Effect of OEB on serum transaminase content. **(E)** Effect of OEB on serum ALP levels. **(F)** H&E staining and **(G)** Oil red O staining of liver tissue sections (scale bar, 50 μm). CON, control group; EtOH, ethanol administration group; OL, low-dose OEB group; and OH, high-dose OEB group. Data are presented as “mean ± SD”. *N* = 8, for all groups. **^##^** EtOH versus CON, *P* < 0.01; * OL or OH versus EtOH, *P* < 0.05; ** OL or OH versus EtOH, *P* < 0.01.

The hepatoprotective effects of OEB were next evaluated in mice. First, liver index (liver w./b.w., %) was calculated in the four mice groups. As shown in Figures 3A,B, liver index and hepatic triglyceride levels were elevated in the EtOH group compared with the control group, whereas pre-treatment with OEB attenuated these increases. Serum ALT, AST, and ALP, which are frequently used to assess liver function, were determined in each experimental group (Figures 3C–E). All three liver biomarkers were elevated by ethanol treatment relative to the control group (*p* < 0.01). Notably, OEB pre-treatment reversed these elevation in a dose-dependent manner. This indicates that 40 μM of OEB may effectively improve alcohol-damaged liver function in mice.

Moreover, the effect of OEB on hepatic histopathological features of ALD mice was examined by both H&E and oil-red O staining. As presented in Figures 3F,G, the hepatic architecture in the control group mice was intact and normal with no lipid vacuoles and no evident inflammatory infiltration, while the acute ethanol exposure dramatically induced visible hepatic histopathological damage including lipid vacuoles and lipid droplets accumulation, inflammatory infiltration and fibrosis in the EtOH group. By contrast, the OEB treatment reversed these hepatic disorders, such as the reduction of steatosis and the exhibition of inflammatory cell, especially in the OH group. These results indicated that the high dose of OEB was more effective than the low dose OEB in relieving ethanol-induced hepatic injury.

### Oenothein B relieves ethanol-induced liver injury by targeting the Keap1/Nrf2 pathway to limit oxidative stress in alcoholic liver disease mice

**FIGURE 4:**
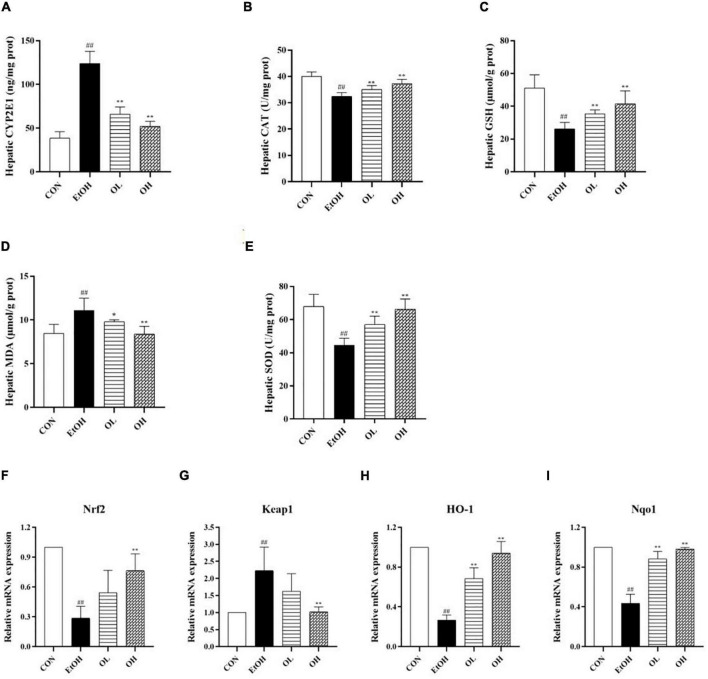
Effect of OEB treatment on hepatic oxidative stress parameters and the expression levels of Keap1/Nrf2 signaling pathway-related genes of ALD mice: **(A)** hepatic CYP2E1 activity; **(B)** hepatic CAT activity; **(C)** hepatic GSH level; **(D)** hepatic MDA activity; **(E)** hepatic SOD activity; **(F)** Nrf2; **(G)** Keap1; **(H)** HO-1; and **(I)** Nqo1. CON, control group; EtOH, ethanol administration group; OL, low-dose OEB group; and OH, high-dose OEB group. Data are presented as “mean ± SD”. *N* = 8, for all groups. **^##^** EtOH versus CON, *P* < 0.01; * OL or OH versus EtOH, *P* < 0.05; ** OL or OH versus EtOH, *P* < 0.01.

Oxidative stress is crucial for the pathogenesis and progression of ALD as it stimulates inflammatory responses and induces apoptosis which promote hepatic tissue injury (26, 27). In order to determine the mechanisms underlying the antioxidant activity of OEB *in vivo*, we measured hepatic indicators - including cytochrome P450 member CYP2E1, catalase (CAT), GSH, MDA, and SOD - in all experimental groups. First, levels of CYP2E1 in the liver were measured, because it was previously shown to be induced by ethanol and plays a crucial role in ROS generation, which aggravates liver injury (28). Hepatic CYP2E1 levels were elevated by ethanol and remarkably reduced by OEB administration (Figure 4A). Furthermore, acute ethanol consumption decreased CAT and MDA activity, decreased GSH levels, and increased the level of MDA (*p* < 0.01) in liver tissues compared with the control group (Figures 4B–E). OEB treatment restored the activity of antioxidant enzymes and the level of MDA in a dose-dependent manner (*p* < 0.01). This suggests that OEB resists ethanol-induced hepatic oxidative stress via elevation of antioxidant enzymes and downregulation of peroxidation in mice.

To identify the role of the Keap1/Nrf2 signaling pathway in OEB-dependent inhibition of ethanol-induced oxidative stress, we measured mRNA expression of genes in this pathway. As shown in Figures 4F–I, ethanol consumption considerably decreased expression of Nrf2, HO-1, and NQO1 and increased expression of Keap1 relative to the control group. Interestingly, OEB treatment significantly inhibited changes in gene expression induced by ethanol (*p* < 0.01).

### Oenothein B amelorates ethanol-induced liver injury by targeting the toll-like receptor 4/nuclear factor-κB pathway to prevent inflammation in alcoholic liver disease mice

**FIGURE 5:**
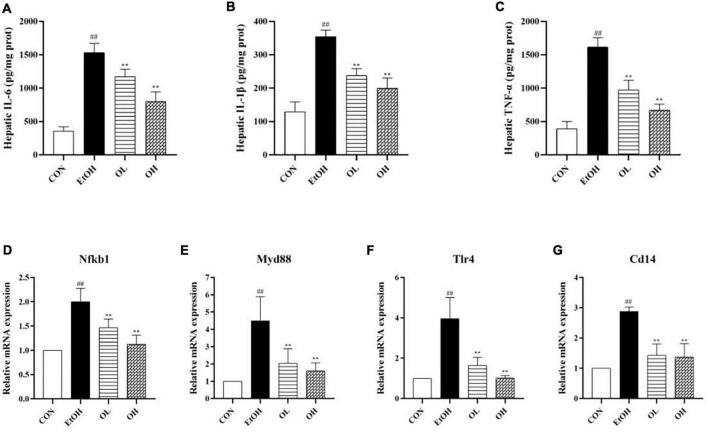
Effect of OEB treatment on hepatic inflammatory indicators and the expression levels of TLR4/NF-κB signaling pathway-related genes of ALD mice: **(A)** serum NO; **(B)** hepatic IL-6 content; **(C)** hepatic IL-1β content; **(D)** hepatic TNF-α content; **(E)** Nfkb1; **(F)** Myd88; **(G)** Tlr4; and **(D)** Cd14. CON, control group; EtOH, ethanol administration group; OL, low-dose OEB group; and OH, high-dose OEB group. Data are presented as “mean ± SD”. *N* = 8, for all groups. **^##^** EtOH versus CON, *P* < 0.01; ** OL or OH versus EtOH, *P* < 0.01.

To assess the mechanism by which OEB alleviates ethanol-induced inflammation, we quantified hepatic pro-inflammatory cytokines including IL-6, IL-1β, and TNF-α. As shown in Figures 5A–C, levels of hepatic IL-6, IL-1β, and TNF-α were significantly increased in the EtOH group compared to the control group (*p* < 0.01). This indicates that ethanol exposure induces substantial inflammation. The OL and OH groups exhibited similar levels of these inflammatory indicators as the control group (*p* < 0.01), indicating that OEB can prevent alcohol-induced inflammation. In particular, the OH group showed greater potential for preventing liver inflammation than the OL group, suggesting that higher OEB doses are more protective from inflammation.

We next determined changes in TLR4/NF-B signaling pathway expression by measuring mRNA levels of related genes in mice livers. When compared with the control group, expression levels of NF-κB, TLR4, Myd88, and CD14 were strikingly increased by ethanol stimulation (Figures 5D–G). OEB intervention dramatically inhibited inflammatory liver necrosis by preventing the overexpression of the TLR4/NF-B inflammatory-related genes (*p* < 0.01).

### Oenothein B alters the structure and composition of gut microbiota in alcoholic liver disease mice

**FIGURE 6:**
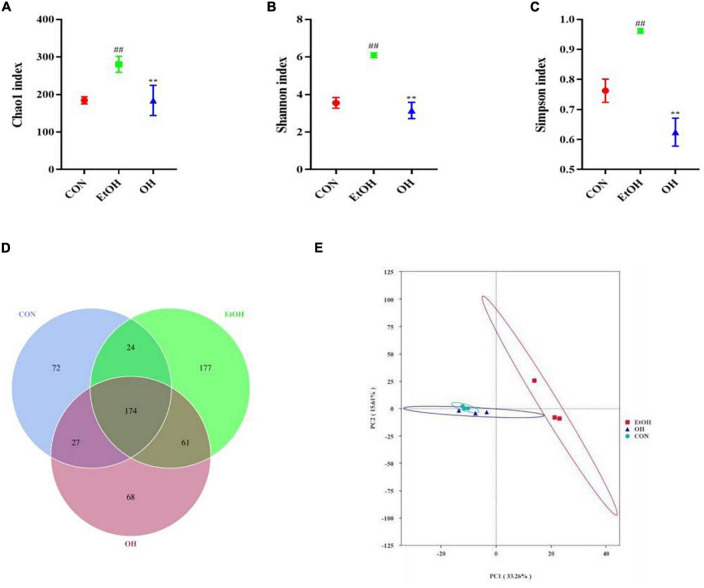
Effect of OEB treatment on α-diversity and β-diversity in gut microbiota. **(A)** Chao1 index; **(B)** Shannon index; **(C)** Simpson index; **(D)** Venn diagram of the number of ASVs of all groups; and **(E)** principal component analysis (PCA) of microbiota. CON, control group; EtOH, ethanol administration group and OH, high-dose OEB group. Data are presented as “mean ± SD”. *N* = 8, for all groups. **^##^** EtOH versus CON, *P* < 0.01; ** OH versus EtOH, *P* < 0.01.

The gut microbiome plays an important role in human health and disease, therefore we examined the effect of ethanol consumption and OEB treatment in ALD mice. We observed that the Chao1 index, Shannon index, and Simpson index were dramatically elevated in the EtOH group compared to the control group (*p* < 0.01) (Figures 6A–C). Moreover, the gut bacterial community α-diversity increased following alcohol intake, whereas OEB treatment prevented this change. Furthermore, there were 72 ASVs in the control group, 177 ASVs in the EtOH group, 68 ASVs in the OH group, 174 ASVs shared by all groups, and just 24 ASVs shared by the control group and the EtOH group (Figure 6D). Consistent with these results, a PCA plot distinctly revealed a shorter distance between the control group and the OH group than between the control group and the EtOH group (Figure 6E). These findings indicate that the microbiome disruption induced by alcohol exposure can be minmized by OEB intervention.

**FIGURE 7:**
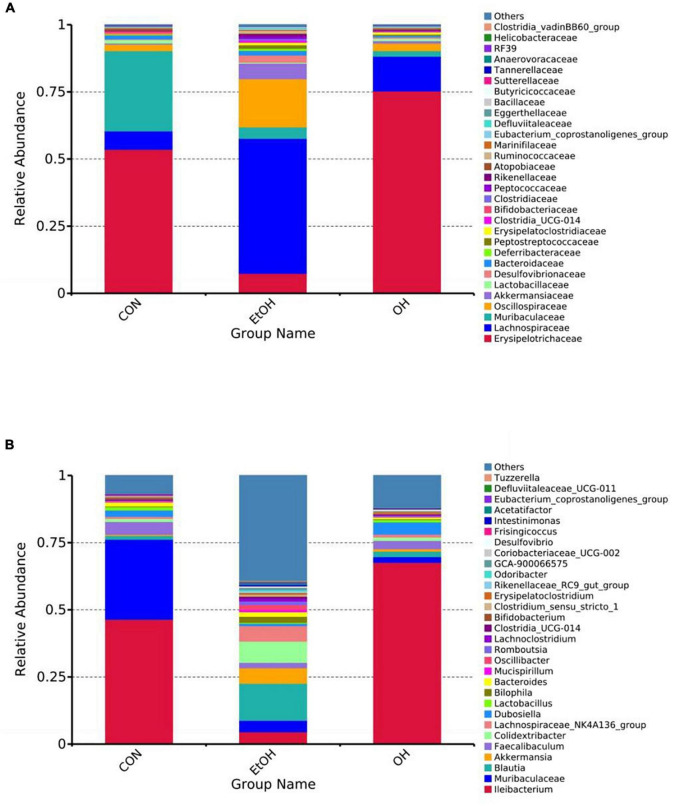
Effect of OEB treatment on gut microbiota. **(A)** Gut microbiota composition at the family level and **(B)** Gut microbiota composition at the genus level. CON, control group; EtOH, ethanol administration group and OH, high-dose OEB group.

Gut microbial composition in all groups were determined based on taxonomic classification analysis. At the family level, the relative abundance of Lachnospiraceae, Oscillospiraceae, and Akkermansiaceae were higher in the EtOH group than the control group, while the relative abundance of Erysipelotrichaceae was lower. The OEB treatment groups had similar levels of these families as in the control group (Figure 7A). At the genus level, we observed increased relative abundance of *Blautia*, *Akkermansia*, *Colidextribacter*, and *Lachnospiraceae_NK4A136_group* and reduced relative abundance of *Ileibacterium*, *Muribaculacea*, and *Faecalibaculum* in the EtOH group in comparison with the control group (Figure 7B). Again, OBE treatment, specifically in the OH group, inhibited these microbial community shifts.

**FIGURE 8:**
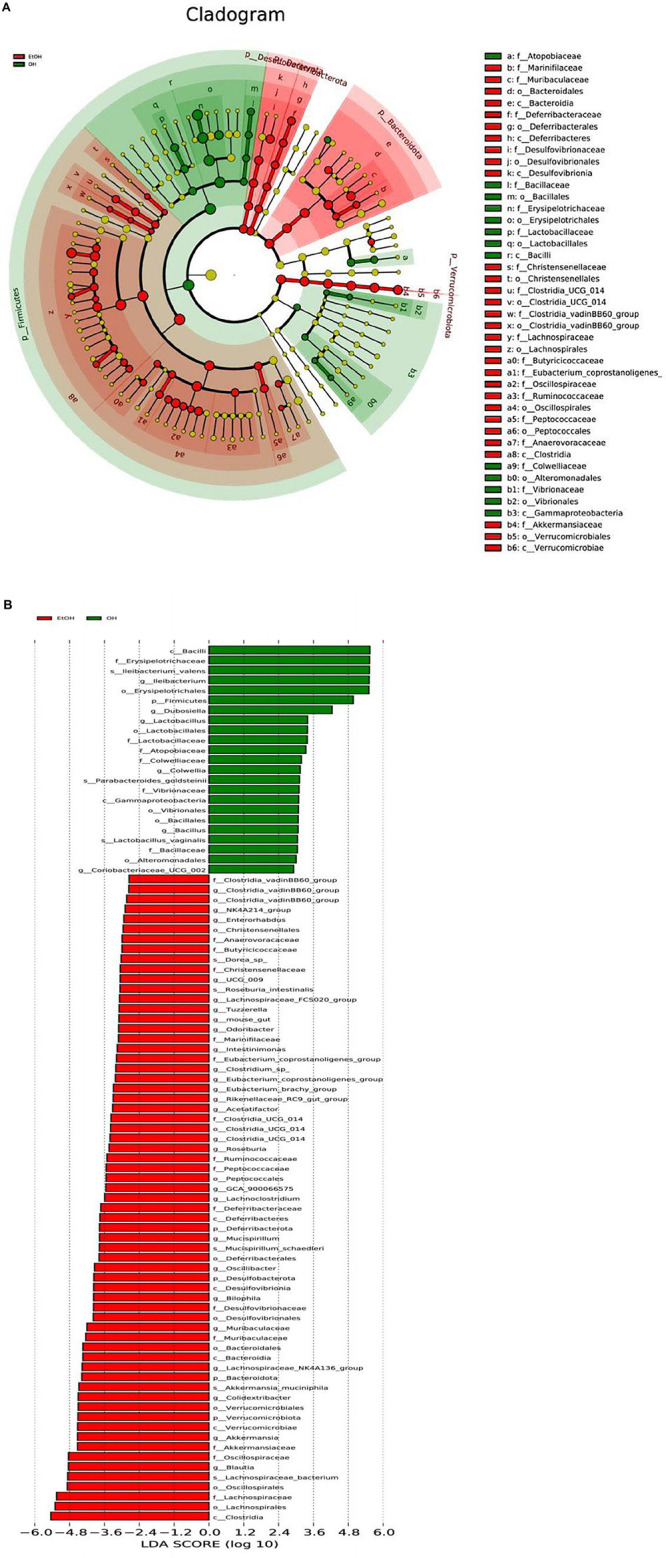
LEfSe analysis of gut microbiota between the EtOH and OH groups. **(A)** Cladogram and **(B)** LDA score. EtOH, ethanol administration group; OH, high-dose OEB group.

Lastly, LEfSe analysis was conducted to illuminate particular distinctions in the predominant bacterial taxa between the OH group and the EtOH group. Results revealed that Clostridia, Lachnospiraceae, Oscillospirales, Blautia, Oscillospiraceae and Akkermansiaceae were more dominant in the EtOH group than in the OH group. *Bacilli*, Erysipelotrichaceae, *Ileibacterium*, Firmicutes, and *Lactobacillus* dominated in the OH group (Figure 8). Taken together, this suggests that OEB supplementation partially prevents alcohol-induced gut microbial dysbiosis.

## Discussion

Prolonged heavy drinking can lead to ALD, a disease that contributes considerably to global morbidity and mortality (29). Though early manifestations of ALD, such as hepatic steatosis and alcoholic hepatitis, are relatively mild and reversible, they can eventually develop into liver fibrosis and sclerosis without abstinence and intervention (30, 31). Inflammation, oxidative stress, lipid accumulation, microbial dysbiosis, apotosis, and tissue regeneration are all important factors that contribute to ALD (4, 32). There are currently few approved therapies for ALD (6), therefore it is necessary to find safe and effective agents that prevent and treat this disease. OEB, a macrocyclic dimer ellagic tannin, has been documented to possess diverse biological effects including antioxidant, anti-inflammation, antitumor, immunomodulatory, and antimicrobial activities (18–23). Many of these processes are directly or indirectly linked with the progression of ALD. Despite this, the hepatoprotective effect of OEB on ALD has not been explored. In the present study, we demonstrated that OEB administration has a protective effect against alcoholic liver injury in HepG2 cells and in mice. This was evidenced by improved hepatic function, alleviation of hepatic oxidative stress, inhibition of inflammation, and restoration of gut microbial dysbiosis following pre-treatment with OEB.

Alcoholic-induced haptic dysfunction is commonly determined by measurement of serum hepatic enzymes levels and liver index, and by observation of histopathological changes (33). Notably, the serum hepatic enzymes ALT, AST, LDH, and ALP are biomarkers of liver damage (34), and tissue H&E and oil-red O staining are a visual reflection of histopathological alterations. In this study, alcohol exposure elevated serum ALT, AST, and ALP levels, TG contents, and liver index in mice (*p* < 0.05). The ALT, AST, and ALP are released from hepatocytes into circulation when hepatic damage occurred. The changes in serum TG contents and liver index clearly demonstrated the lipid metabolism disorder induced by alcohol. However, OEB treatment reversed this elevation. Consistent with *in vivo* results, OEB significantly reduced elevation of ALT, AST, and LDH in cell supernatant (*p* < 0.05), and inhibited ethanol-induced cell death to an extent. Histopathological examination of the EtOH group revealed lipid vacuoles, lipid droplet accumulation, inflammatory infiltration, and fibrosis, and OEB treatment alleviated these pathological damages. Overall, the above findings revealed that the OEB supplementation might be useful in maintaining the hepatocyte integrity and regulating the lipid metabolism in hepatocyte to improve liver dysfunction and pathological damage induced by alcohol both *in vivo* and *in vitro*. Similar hepatoprotective effects have been observed in ellagic acid, an analogue of OEB (35).

Numerous previous studies have reported that CYP2E1 plays a vital role in the development of ALD (36, 37). Excessive alcohol intake activates CYP2E1, which not only induces the formation of acetaldehyde, but also results in increased ROS generation and oxidative damage (11, 28). The Keap1/Nrf2 signaling pathway is an important component of the hepatic antioxidant system, and the protective effects of the Keap1/Nrf2 signaling pathway against oxidative stress have been identified in a number of ALD models (38, 39). For example, past relevant studies have reported that quercetin may inhibit alcohol-induced hepatic oxidative stress in zebrafish through mediation of the Keap1/Nrf2 signaling pathway (40). Keap1 binds Nrf2 and enables its ubiquitination and destruction in the cytoplasm in baseline conditions which results in persistently low levels of Nrf2. However, when hepatocytes are subjected to overwhelming oxidative stress, Nrf2 separates from Keap1 and moves into the nucleus. There, it forms heterodimers with the small Maf proteins (MafF, MafG, and MafK) and binds to antioxidant response elements (AREs), which in turn control the expression of downstream target genes like HO-1, NQO1, SOD, CAT, and GSH (41). Activated antioxidant enzymes and nonenzymatic antioxidants work synergistically to reduce oxidative stress injury. In this work, supplementation with OEB increased gene expression of Nrf2, HO-1, and NQO1, and reduced Keap1 levels. Moreover, we observed elevated levels of SOD, CAT, and GSH and decreased levels of MDA and CYP2E1 in the OL and OH groups, implying that OEB substantially ameliorates hepatic oxidative stress. In agreement with our *in vivo* study, SOD, CAT, and GSH levels were significantly lower in the OL and OH group HepG2 cells, whereas MDA and ROS levels exhibited the opposite trend. These results indicate that OEB improves alcohol-induced liver injury through regulation of the Keap1/Nrf2 signaling pathway, which relieves oxidative stress.

In addition to the hepatic oxidative stress, ALD pathogenesis also depends on gut-derived LPS-mediated liver inflammation (42, 43). Acute alcohol consumption has been shown to increase intestinal permeability leading to LPS entering the portal vein and reaching the liver (44, 45). This leaked LPS activates Kupffer cells’ toll-like receptor 4 (TLR4), which in turn increases expression of downstream genes such as nuclear factor-B (NF-B), Myd88, and CD14 (46, 47). Activation of these inflammatory genes results in the production of adverse inflammatory cytokines that trigger liver inflammation and injury (48). Our results suggested that OEB intervention significantly downregulated expression of TLR4 and its downstream inflammatory mediators, which also led to significant decreases in TNF-α, IL-1β, and IL-6 levels in mice livers relative to the EtOH group. These results are in agreement with a previous study by Xia et al. (49). OEB also inhibited inflammation *in vitro* as shown by the reduction of ethanol-induced NO levels in cell supernatant of HepG2 cells. Previous studies have shown that OEB restrains p65 nuclear translocation, thereby decreasing NF-κB binding activity (50) and inhibiting TLR/NF-κB-dependent NO synthesis (51), which aligns with our findings. Taken together, OEB resists LPS-mediated liver inflammation by targeting the TLR4/NF-κB signaling pathway.

There is growing evidence that enteric dysbiosis is linked to the progression of ALD (13, 52). Interestingly, recent studies of acute ethanol intake in humans and mice reported enteric dysbiosis with both numerical and proportional perturbations (53). Therefore, we speculated that treatment with OEB would modulate gut microbiota that are disrupted by alcohol intake and subsequently decrease liver injury, thus providing insight into the mechanism of OEB. To investigate this, we detected the gut microbial composition in each group by sequencing bacterial 16S rRNA.

Measurement of α-diversity indicated that acute alcohol intake decreases the richness and diversity of intestinal microbiota in mice, which is in line with previous studies by Li et al. (54). Specifically, the Chao1 index, Shannon index, and Simpson index were higher in the in EtOH group mice than in the control group mice. OEB intervention reversed this trend. β-diversity analysis demonstrated that the gut microbiota among the OH groups were distinct from the EtOH group and relatively close to the control group. This analysis also demonstrated that the gut microbiome of OEB treated mice were more closely aligned with the control group compared to the EtOH group, implying that OEB administration may restore the alcohol-disrupted microbiome composition. This is consistent with research by Ferrere et al. (55).

Alcohol abuse alters the composition and abundance of gut microbiota, leading to abnormalities in the gut-liver axis (12, 56). As described in our results section, the EtOH group had increased relative abundance of Lachnospiraceae, Oscillospiraceae and Akkermansiaceae and reduced relative abundance of Erysipelotrichaceae compared with the control group. A previous investigation found elevated abundance of Lachnospiraceae in humans with alcohol liver injury (57). Increased abundance of Oscillospiraceae in mice with metabolic syndrome, induced by high fat and cholesterol diet, has also been reported (58). As a mucin-degrading intestinal bacterium, Akkermansiaceae can alter intestinal barrier function by inducing inflammation and promoting colonic tumorigenesis (59). A previous study showed an enriched abundance of Akkermansiaceae in mice with colorectal cancer (60). In addition, Erysipelotrichaceae is known to produce butyric acid, which is a primary energy source within the colonic epithelium that is vital to intestinal health (61). Decreased production of butyrate has been associated with human disease (62). Of note, OEB supplementation restored the changes in above bacterial families induced by alcohol administration.

Some potentially beneficial bacterial genera - such as *Ileibacterium, Muribaculacea, and Faecalibaculum* - that were reduced in the EtOH group were restored in OEB treatment groups. The relative abundance of *Ileibacterium* has been reported to be reduced in the context of a metabolic disorder induced by chronic alcohol intake (63). Furthermore, a recent study has suggested that *Muribaculacea* positively correlates to colonic inner mucus layer formation and barrier function (64), and its abundance was strikingly related to the concentration of propionate (65). *Faecalibaculum* is considered as a critical component of carbohydrate and energy metabolism pathways in the host (66), and it has been reported to be abundant in mice with metabolic disorders (67). However in our study, relative abundance of *Blautia, Akkermansia, Colidextribacter, and Lachnospiraceae_NK4A136_group* was elevated by alcohol consumption. It has been reported that patients with liver fibrosis possess a greater abundance of genus *Blautia* (68). *Akkermansia* is Gram-negative bacteria whose cell wall component LPS is known to induce hepatic inflammation via the gut-liver axis (69). Wang et al. found that *Colidextribacter* had a positive correlation with serum MDA and a negative correlation with serum SOD and GPx levels (70), which accelerated the oxidative stress in body. As a pathogenic bacterium, *Lachnospiraceae _NK4A136_group* may be associated with intestinal disorders (71). In the present study, OEB treatment regulated these genera, keeping their abundance at a consistent level with the control group. Ultimately, OEB may improve alcohol-induced gut dysbiosis by modulating composition and relative abundance of the intestinal microbiome.

Interestingly, we observed that OEB supplementation specifically elevated the abundance of SCFA (short chain fatty acid) producers (*Muribaculaceae, Erysipelotrichaceae*) in the gut of alcohol-induced mice, which may have contributed to the increased levels of SCFAs in the intestine. SCFAs are metabolites of the intestinal microbiota, and according to a previous study, they are involved in activation of the Nrf2 signaling pathway in the liver which enhances the hepatic antioxidant system (72). Additionally, OEB intervention decreased the abundance of Gram-negative bacteria (*Akkermansia*), whose cell wall component LPS activates the TLR4/NF-κB signaling pathway in the liver and causes inflammation. Therefore, we speculate that OEB may ameliorate alcohol-induced liver disease via regulation of the gut-liver axis. Further studies are required to systemically investigate the mechanism by which this occurs.

## Conclusion

This research revealed that OEB treatment can efficiently prevent acute alcohol-induced liver injury by activating the Keap1/Nrf2 signaling pathway which resists oxidative stress, and by alleviating hepatic inflammation via downregulation of the TLR4/NF-κB signaling pathway. Moreover, regulation of gut dysbiosis was also found to be a hepatoprotective mechanism of OEB. Together, these findings provide a theoretical basis and research approach for the application of OEB as a preventative and treatment agent for ALD.

## Data availability statement

The data presented in this study are deposited in the jianguoyun repository, accession links: https://www.jianguoyun.com/p/DT9reqQQ1oOACxiWvdsEIAA, https://www.jianguoyun.com/p/DdVrGAgQ1oOACxibvdsEIAA, https://www.jianguoyun.com/p/DTz_SOIQ1oOACxidvdsEIAA, https://www.jianguoyun.com/p/DYuM7QkQ1oOACxifvdsEIAA, https://www.jianguoyun.com/p/Dd-0tV8Q1oOACxihvdsEIAA, https://www.jianguoyun.com/p/DXLyei8Q1oOACxijvdsEIAA, and https://www.jianguoyun.com/p/DVOiIqwQ1oOACxilvdsEIAA.

## Ethics statement

This animal study was reviewed and approved by the Laboratory Animal Center of South China Agricultural University (SYXK 2014-0136).

## Author contributions

LX: data curation, formal analysis, investigation, writing—review and editing and validation. WL: data curation, formal analysis, investigation and roles, and writing—original draft. S-YC: data curation, formal analysis, and writing—review and editing. X-WD: curation, formal analysis, and investigation. W-FD: data curation and investigation. GL: data curation and formal analysis. Y-JC: writing—review and editing. YC: investigation, writing—review and editing, and supervision. All authors contributed to the article and approved the submitted version.
